# Performance of the SAPS 3 admission score as a predictor of ICU mortality in a Philippine private tertiary medical center intensive care unit

**DOI:** 10.1186/2052-0492-2-29

**Published:** 2014-04-24

**Authors:** Aaron Mark R Hernandez, Jose Emmanuel M Palo

**Affiliations:** Section of Adult Critical Care Medicine, Department of Medicine, The Medical City, Pasig City, Philippines

**Keywords:** Severity of illness, SAPS 3, Intensive care unit, Critical care, Outcome assessment, ICU mortality, Quality of care

## Abstract

**Background:**

This study aimed to assess the performance of the Simplified Acute Physiology Score 3 (SAPS 3) as a predictor of ICU mortality in critically ill patients of different case mixes admitted to an intensive care unit.

**Methods:**

This retrospective cohort study was performed from January 2011 to August 2013 in the intensive care unit of a private tertiary referral center in the Philippines. Predicted ICU mortality was calculated using the SAPS 3 global model. Observed versus predicted mortality rates were compared, and the standardized mortality ratio (SMR) was calculated. The discrimination and calibration characteristics of the SAPS 3 system to predict ICU mortality were assessed.

**Results:**

A total of 2,426 patients were included. The observed ICU mortality was 277 (11.42%). The SAPS 3 global model had fair to good discrimination with an area under the receiver operating characteristic curve of 0.80 (CI 0.78–0.81). Good calibration was seen with the Hosmer-Lemeshow goodness of fit at *Ĉ* = 11.51 (*p* = 0.175). Standardized mortality ratio was 0.36 (0.26–0.81).

**Conclusion:**

The global SAPS 3 prediction model showed fair to good discrimination and good calibration in predicting mortality in our intensive care unit. Different levels of discrimination and calibration across the different subgroups analyzed suggest that overall ICU performance seemed to be affected by case mix variations.

**Electronic supplementary material:**

The online version of this article (doi:10.1186/2052-0492-2-29) contains supplementary material, which is available to authorized users.

## Background

A critical care program or unit can be assessed using a variety of severity scoring systems or models that allow the estimation of mortality probabilities and comparison with actual mortality rates. Resource allocation and quality improvement strategies could then follow from these severity-adjusted mortality estimates [1, 2].

However, reliability of a severity score reportedly deteriorates when applied to different populations, probably due to case mix, the level and quality of care, and the development of new treatment options changing overall patient outcomes [2]. Application of a severity scoring system in the intensive care unit with different case mixes raises issues of the system's reliability and validity [1].

The Simplified Acute Physiology (SAPS) 3 admission score is one of these models used to predict hospital mortality from admission data taken within the first hour of the patients' admissions. From this score, global and region-specific equations for hospital mortality have been derived [3]. The performance of this model has shown mixed results among different case mixes in different studies [3].

One basic tool is the calculation of a standardized mortality ratio (SMR) between observed and scoring system-predicted mortality rates. An SMR of lower than 1, for instance, suggests ICU performance to be better than the reference ICUs used to develop the scoring system. But SMRs that are consistently and significantly less than 1 raise questions regarding the reliability of the scoring system to predict mortality for that particular ICU's patient population.

Severity scoring system reliability can be quantified in terms of calibration, which represents the level of accordance between observed and predicted probabilities of the outcome [4]. This is derived from tests such as the Hosmer-Lemeshow ‘goodness-of-fit’ test [5] or the calibration belt [4]. Discrimination, another essential quality, is quantified with measures such as sensitivity, specificity, and more completely, the area under the receiver operating characteristic curve (AUROC) [5]. An AUROC of 0.5 indicates that the model does not predict better than chance. The discrimination of a prognostic model is considered perfect if AUROC = 1, good if AUROC > 0.8, moderate if AUROC is between 0.6 and 0.8, and poor if AUROC < 0.6 [6].

This study aims to assess the performance of the SAPS 3 in its ability to predict ICU mortality among critically ill patients of different case mixes admitted to a Philippine intensive care unit from 2011 to 2013. Trends on the quality of care delivered in our intensive care unit as a whole and across different disease conditions can be extracted.

## Methods

This study was conducted in the Intensive Care Unit of The Medical City, an 18-bed mixed medical and surgical unit serving all adult (> 19 years old) critically ill patients from all departments of the institution. The unit is served by the Section of Adult Critical Care Medicine with its complement of staff intensivists, fellows-in-training, and rotating residents.

This is a retrospective cohort study. Data was collected from all ICU admissions from 1 January 2011 to 31 August 2013. Patients younger than 19 years old were excluded from the study. All adult patients 19 years of age and above, regardless of diagnosis, including patients admitted for post-operative monitoring, burns, and trauma, were included. Only the first ICU admission of patients with multiple ICU admissions during a single hospital stay was considered. Patients with missing components for SAPS 3 analysis were excluded. The study was approved by The Medical City Institutional Review Board.

Data was taken from a database of previously collected SAPS 3 scores of all ICU patients admitted during the stated period. A standardized data collection form was used (see Additional file 1), which included all components of the SAPS 3 score described by the original SAPS 3 [7]. All data was collected by rotating medical residents and was screened and processed by the critical care fellows to formulate the predicted mortality rates based on the SAPS 3 severity score, using the general formula recommended by Moreno et al. [7] translated to Microsoft Excel format. Actual mortality rates were taken and compared to predicted rates, and the ICU SMR was computed by dividing the observed ICU mortality by the predicted mortality.

Statistical analysis was done using Microsoft Excel 2010 for Windows and Macintosh for computation of the SMR. MedCalc Software 12.3.0 (MedCalc Software, Belgium) was used to perform the rest of the statistical analyses. A *p* value of less than 0.05 was set as statistically significant. Discrimination was determined by analysis of the area under a receiver operating characteristic curve (AUROC) using the method described by Hanley and associates [8]. Calibration was assessed using the Hosmer-Lemeshow goodness-of-fit statistics [9] and by review of the SMRs. In the analysis, lower Hosmer-Lemeshow *Ĉ* values and a *p* value of more than 0.05 would indicate a good fit of the model. The 95% confidence interval (CI) for the SMR was calculated using an online SMR analysis calculator [10], taking Fisher's exact CI [11].

## Results

### Baseline characteristics of patients

There were 2,632 distinct admissions during the study period. A total of 2,426 (92.2%) patients were included in this study. Two hundred and six patients were excluded: 1 patient for age < 19 years, 46 (1.9%) for readmission to the ICU during the same hospital admission, and 159 (6.6%) for incomplete SAPS 3 data. ICU mortality during this period was 277 (11.4%). The majority (2,123, 87.5%) of the cases admitted to the ICU were medical cases, with pneumonia (615, 25.3%) and sepsis syndrome (462, 19%) as the most frequent primary diagnoses. Baseline characteristics of the included patients are shown in Table 1.

**Table 1:** **Basic demographic characteristics**

Variables	Characteristics
Age (years, mean, ±SD)	62 (17)
> 65 years [*n* (%)]	1,077 (44.39)
≤ 65 years [*n* (%)]	1,349 (55.61)
Sex [*n* (%)]	
Male	1,450 (59.77)
Female	976 (40.23)
Type of patients [*n* (%)]	
Medical	2,123 (87.51)
Acute coronary syndrome	371 (15.29)
Post-cardiac arrest	131 (5.39)
Shock, all types	341 (14.06)
Cardiogenic	30 (1.24)
Sepsis and septic shock	462 (19.04)
Acute respiratory failure	305 (12.57)
Pneumonia, all types	615 (25.35)
Solid tumor	167 (6.88)
Hematologic malignancies	15 (0.62)
Cerebrovascular accidents	96 (3.96)
GI bleeding	80 (3.29)
Surgical	303 (12.49)
Coronary artery bypass graft (CABG)	115 (4.74)
Non-CABG surgeries	188 (7.75)
SAPS 3 scores	55 (16–124)
SAPS 3 predicted mortality (%), mean ± SD	31 ± 26.33
Observed ICU mortality [*n* (%)]	277 (11.42)

### Calibration of SAPS 3 scores

**Figure 1:**
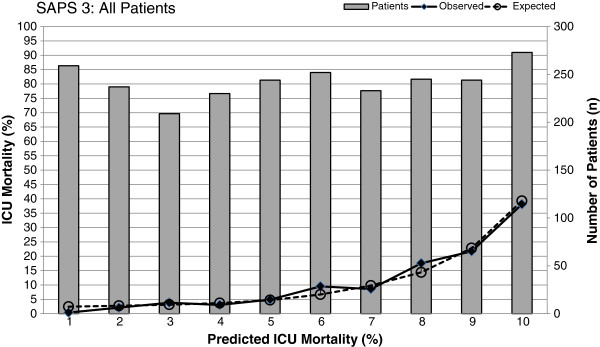
**Calibration curve for global SAPS 3 model comparing actual and predicted ICU mortality.** Hosmer and Lemeshow test for goodness of fit showed good calibration (*Ĉ* = 11.51, *p* = 0.175).

The global SAPS 3 model exhibited satisfactory calibration for the entire population (*Ĉ* = 11.5, *p* = 0.18). The uniformity of fit of the model was consistent along the deciles in the calibration curve (Figure 1). Subgroup analysis showed that the global SAPS 3 model showed good calibration for age > 65 years (*Ĉ* = 6.1, *p* = 0.64), all medical conditions treated as a group (*Ĉ* = 9.4, *p* = 0.31), and all surgical conditions treated as a whole (*Ĉ* = 4.8, *p* = 0.78), as shown in Table 2. Poor calibration was noted with patients aged ≤ 65 years (*Ĉ* = 19.2, *p* = 0.01) and patients admitted with solid tumors (*Ĉ* = 22.3, *p* = 0.004).

**Table 2:** **Calibration and discrimination of SAPS 3 and SMRs for all patients and various subgroups**

	Number	Actual mortality	Predicted mortality (mean ± SD)	Goodness-of-fit test^a^		AUROC^b^	SMR
		[***N*** (%)]		***Ĉ***	***p***	(95% CI)	(95% CI)
All	2,426	277 (11.42)	31.61 ± 26.3	11.51	0.18	0.80 (0.78–0.81)	0.36 (0.26–0.81)
Age > 65 years	1,077	141 (13.09)	40.92 ± 25.6	6.08	0.64	0.77 (0.74–0.79)	0.32 (0.19–0.54)
Age ≤ 65 years	1,349	136 (10.08)	24.18 ± 24.4	19.21	0.01	0.82 (0.80–0.84)	0.42 (0.23–0.76)
Medical	2,123	259 (12.19)	33.81 ± 26.3	9.44	0.31	0.79 (0.77–0.80)	0.36 (0.21–0.62)
Acute coronary syndromes	371	34 (9.16)	23.70 ± 23.1	4.81	0.78	0.79 (0.74–0.83)	0.39 (0.20–0.72)
Post-cardiac arrest, all causes	131	47 (35.88)	63.81 ± 20.9	12.25	0.14	0.66 (0.57–0.74)	0.56 (0.40–0.76)
Sepsis and septic shock	462	90 (19.48)	47.65 ± 25.2	6.95	0.54	0.69 (0.65–0.74)	0.41 (0.26–0.62)
Shock, all types	341	88 (25.81)	51.56 ± 26.3	2.79	0.95	0.68 (0.63–0.73)	0.50 (0.33–0.72)
Acute respiratory failure, all causes	305	41 (13.44)	46.89 ± 23.2	6.93	0.54	0.75 (0.69–0.79)	0.29 (0.16–0.47)
Pneumonia, all types	615	101 (16.42)	46.92 ± 24.1	3.95	0.86	0.72 (0.69–0.76)	0.35 (0.21–0.55)
Acute respiratory distress syndrome	20	7 (35)	51.45 ± 26.0	4.06	0.85	0.93 (0.74–1.00)	0.68 (0.47–0.95)
Solid tumors	167	30 (17.96)	40.14 ± 28.8	22.28	0.004	0.81 (0.74–0.87)	0.45 (0.27–0.68)
Hematologic malignancies	15	6 (40)	60.00 ± 23.6	7.15	0.21	0.61 (0.33–0.84)	0.67 (0.48–0.91)
Acute kidney injury	93	16 (17.20)	46.90 ± 23.7	6.66	0.57	0.79 (0.69–0.86)	0.37 (0.23–0.58)
Encephalopathy, all types	80	12 (15)	46.15 ± 26.7	4.97	0.76	0.70 (0.58–0.80)	0.33 (0.18–0.54)
Upper GI bleeding	80	8 (10)	31.73 ± 22.3	3.96	0.86	0.82 (0.72–0.90)	0.32 (0.15–0.58)
Cerebrovascular accidents	96	8 (8.33)	26.08 ± 20.5	2.52	0.93	0.82 (0.73–0.89)	0.32 (0.16–0.60)
Surgical	303	18 (5.94)	16.19 ± 21.2	4.84	0.77	0.86 (0.82–0.90)	0.37 (0.14–0.72)
Coronary bypass grafting (CABG)	115	6 (5.22)	9.41 ± 18.1	9.74	0.08	0.85 (0.78–0.91)	0.55 (0.23–1.24)
Non-CABG surgery	188	12 (6.38)	20.34 ± 21.9	1.98	0.98	0.91 (0.87–0.95)	0.31 (0.14–0.64)

^a^Computed using the Hosmer and Lemeshow goodness-of-fit *Ĉ* test. ^b^Analyzed using the method by Hanley and associates.

### Comparison of discrimination

The discriminative power of the SAPS 3 model was fair to good for the whole population (AUROC = 0.80, 0.78–0.81, Table 2). The SAPS 3 model exhibited fair to good discrimination for medical cases (AUROC = 0.79, 0.77–0.80) with different discriminatory patterns noted, from poor to fair for hematologic malignancies (AUROC = 0.61, 0.33–0.84) and post-arrest syndrome of all causes (AUROC = 0.66, 0.57–0.74) to good to very good for groups like acute respiratory distress syndrome (ARDS) (AUROC = 0.93, 0.74–1.0) and patients with solid tumors (AUROC = 0.81, 0.74–0.87). The model showed better discrimination for surgical cases, with good discriminatory power (AUROC = 0.86, 0.82–0.90), even for its subgroups: CABG (AUROC = 0.85, 0.78–0.91) and non-CABG surgery (AUROC = 0.92, 0.87–0.95).

### Standardized mortality ratio

The SAPS 3 model consistently significantly overestimated ICU mortality for our ICU, with an SMR for all included patients at 0.36 (0.26–0.81). This was true for both medical (SMR = 0.36, 0.21–0.62) and surgical (SMR = 0.37, 0.14–0.72) populations. Trends to exactly estimating ICU mortality were seen with patients admitted with ARDS (SMR = 0.68, 0.47–0.95), hematologic malignancies (SMR = 0.67, 0.48–0.91), and patients who underwent CABG (SMR = 0.55, 0.23–1.24, Table 2).

## Discussion

The study evaluated the accuracy of the SAPS 3 mortality prediction model when used in a local tertiary center's intensive care unit. It is important to validate the performance of the model, in this case SAPS 3, prior to application to other centers [2] and before its use to make quality of care assessments [12]. This study includes the largest Philippine cohort of ICU patients in which the SAPS 3 model was used.

The model showed good calibration in our study population, and this was true for almost all subgroups analyzed, except for patients aged less than or equal to 65 years old and for patients with solid tumors.

The study showed that the SAPS 3 global model had fair to good discriminative power. This was lower than other external validation studies using SAPS 3 (0.82 to 0.93 in previous studies) [2, 3, 13, 14]. Analysis of the discrimination patterns for the subgroups in our study showed lower scoring system discrimination (AUROC 0.66–0.93) than in the study of a Thai intensive care unit (AUROC 0.89–0.96) where subgroup analysis (for age, diagnosis, sex, etc.) was made. The SAPS 3 discriminatory pattern for subpopulations was good to very good in their ICU compared to poor to good in ours [2].

This overall pattern of good calibration with fair discriminatory power is reported when an existing severity score system is applied in a population different from the reference ICU population from which the score equation was developed [15, 16]. Our SAPS 3 validity pattern is similar to that reportedly seen in a Korean intensive care unit [16]. This supports the observation that the original SAPS 3 database possibly does not represent a global case mix, especially as specific geographic regions or patient diagnoses were underrepresented [1]. This, however, does not limit the use of the model in predicting mortality, even in our population.

Evidence of different levels of calibration and discrimination on subgroup analysis supports that the global SAPS 3 model was indeed affected by differences in case mix [1], where the overall fair to good discrimination may have been affected by subgroups with a wide range of discrimination characteristics.

Our study showed that the SAPS 3 score significantly overestimated the actual ICU mortality, with an SMR very much less than 1, and this was consistently seen across all analyzed subgroups as shown in Table 2. Low SMRs (less than 1) suggest adequacy of resource allocation, decreases in lead-time bias, proper staffing, and the availability of appropriate technology [17]. Differences in SMRs have been ascribed to unmeasured different factors including differences in intensive care provision, the presence of structures and processes inherent to the healthcare system, resource limitations, cultural differences, and genetic predispositions [14]. Low SMRs were reported in our ICU from 2011 to 2013, reflecting the consistent delivery of intensive care.

There are several limitations to our study. First, this is a retrospective study; another issue is that the study derived its data from a single center ICU, limiting the sample size as well as the case mix included in the study compared to the original SAPS 3 cohort and affecting generalizability even within our country. Our subgroup analyses had smaller samples that make the statistical analysis less robust, with wider confidence intervals. The last limitation is one inherent to the Hosmer-Lemeshow goodness-of-fit test, which depends on the sample size, such that small samples tend to give a better fit and larger samples lead to poorer fit, as we have shown.

## Conclusion

The global SAPS 3 prediction model showed fair to good discrimination and good calibration in predicting mortality in our intensive care unit. Different levels of discrimination and calibration across the different subgroups analyzed suggest that overall ICU performance is affected by case mix variations. A low SMR through the 32-month study period suggests good allocation and delivery of intensive care in our center. It is recommended that this model be tested in other centers and that a consolidated database be formed. A customized model of the current SAPS 3 prediction tool can then be formulated for better representation of the Philippine intensive care population.

## Authors’ information

AMRH is the chief fellow of the Adult Critical Care Medicine Fellowship Training of The Medical City from 1 April 2013 to 31 March 2014. He is a member of the rapid response team committee of the hospital and has participated in projects on transport of the critically ill and improvement of pharmacy involvement in the response team. He has been active in researches in critical care quality-of-care studies, including international ICU nutrition surveys. His current interests include ICU nutrition, clinical pharmacy in the ICU, and clinical toxicology.

JEMP is the head of the Section of Adult Critical Care Medicine of the Department of Internal Medicine of The Medical City, and Consultant Director of the ICU. He is the Philippine representative and site coordinator for numerous international critical care research collaborations including the Intensive Care Over Nations (ICON) survey, Fluid Challenges in Intensive Care Trial (FENICE), and the upcoming LungSafe study among others. He is a member of the Asian Critical Care Clinical Trials Group.

## Electronic supplementary material

Additional file 1: **SAPS-3 Admission Score – The Medical City Patient Data Sheet.** A copy of the standardized data collection form. (PDF 538 KB)
