# Supplementary material for: Performance of the SAPS 3 admission score as a predictor of ICU mortality in a Philippine private tertiary medical center intensive care unit
Source: J Intensive Care. 2014 Apr 24;2(1):29. doi: 10.1186/2052-0492-2-29 (PMC4267583; doi:10.1186/2052-0492-2-29)
Supplement: Supplementary file 1 — Additional file 1: SAPS-3 Admission Score – The Medical City Patient Data Sheet. A copy of the standardized data collection form. (PDF 538 KB) [file 40560_2014_36_MOESM1_ESM.pdf]

SAPS-3 ADMISSION SCORE – The Medical City Patient Data Sheet

(Reminders: Please fill in the required data as completely as possible during the ***first hour of ICU admission****. Direct all questions to any of the Critical Care Medicine Fellows. Submit forms to any of the CCM fellows. Thank you!)*

Date/Time of ICU admission ________________
Name (Initials): ___________________________
Age/Sex: ___________ **PIN:** _______________
Admitting diagnoses:

☐ **Length of stay <6hrs (check if yes)**
ICU Resident-in-charge: ___________________

(Scoring c/o CCM Fellow:)
SAPS 3 Admission score: ______________
Predicted Mortality: ______________

I. Condition of patient before ICU admission

1. Length of stay hospital stay before ICU admission: ________days
2. Intra-hospital location before ICU admission:
   ☐OR ☐ER ☐Other ICU (i.e. ACSU, NICU, Telemetry, Recovery Room) ☐Others (i.e Wards)
3. Co-morbidities
   - ☐ Cancer therapy (i.e. chemotherapy, immunosuppresion, radiotherapy, steroid treatment)
   - ☐ metastatic cancer
   - ☐ hematological cancer (lymphoma, acute leukemia, multiple myeloma)
   - ☐ Chronic heart failure (NYHA IV)
   - ☐ Cirrhosis
   - ☐ AIDS
4. Use of major therapeutic options (i.e vasopressors) before ICU admission: ☐ yes ☐no

II. Condition of patient at ICU admission

1. ICU admission:
   ☐ planned ≥ 12 hours in advance ☐ unplanned or planned < 12 hrs in advance
2. Reason for ICU admission
   CVS: ☐ Rhythym disturbance ☐Hypovolemic shock ☐septic shock
   ☐ anaphylaxic shock, mixed type of shock, & other types of shock
   Hepatic: ☐ Liver failure
   Digestive: ☐ Severe pancreatitis ☐ Acute abdomen ☐ Others
   Neurologic: ☐ Intracranial mass effect ☐focal neurologic deficit ☐ seizure
   ☐coma, stupor, obtunded patient, agitation, vigilance disturbances, confusion, delirium
   ☐none of the above; kindly write down reason for admission: ___________________________________
3. Surgical status at ICU admission: ☐ scheduled surgery ☐ emergent surgery ☐ no surgery
4. Anatomical site of surgery:
   - ☐ Transplantation surgery
   - ☐ Trauma
   - ☐ Cardiac (CABG w/o valve repair)
   - ☐ Neurologic (CVA accident)
   - ☐ Others: kindly indicate anatomical site ____
   - ☐ Not applicable / no surgery
5. Acute infection at ICU admission:
   a. nosocomial infection? ☐ yes ☐ no
   b. respiratory (i.e. pneumonia, bronchitis, lung abscess, other lower RTI)? ☐ yes ☐ no

III Physiologic Derangement at ICU admission

1. Estimated GCS (lowest) ____________
2. Total bilirubin (highest) ____________ mg/dL
3. Body Temp (highest) ____________ °C
4. Creatinine (highest) ____________ mg/dL
5. Heart rate (highest) ____________ beats/minute
6. Leucocytes (lowest) ____________ x 10^9/L
7. pH (lowest) ____________
8. platelet (lowest) ____________ x 10^9/L
9. Systolic BP (lowest) ____________ mm Hg
10. Oxygenation status: PaO_2 ____________mm Hg, FiO_2 ____________%, P/F Ratio ____________

    ☐check if px is on mechanical ventilator
